# Multilevel modeling analysis of bottle feeding and its determinants among children 0–23 months in East Africa: evidence from recent DHS data (2015–2022)

**DOI:** 10.1186/s13006-024-00629-w

**Published:** 2024-04-08

**Authors:** Bewuketu Terefe, Adane Habtie, Bogale Chekole

**Affiliations:** 1 Department of Community Health Nursing, School of Nursing, College of Medicine and Health Sciences, University of Gondar, Gondar, Amhara region Ethiopia; 2 Department of Public Health, College of Medicine and Health Sciences, Wolkite University, Southern, Ethiopia; 3 Department of Comprehensive Nursing, College of Medicine and Health Sciences, Wolkite University, Southern, Ethiopia

**Keywords:** Bottle feeding, East Africa, Multilevel modeling

## Abstract

**Background:**

Despite breastfeeding recommendations, the prevalence and length of breast milk feeding in developing nations is rapidly decreasing, with bottle feeding taking its place. This reduces the effectiveness of breastfeeding and is associated with diarrheal disease mortality and morbidity. The purpose of this study was to determine the prevalence, distribution, and determinants of bottle feeding among under-two-year-old children in the region.

**Methods:**

The ten East African countries’ Demographic and Health Surveys (DHS) recent data from 2015 to 2022 was used. The data were weighted using sample weights for probability sampling and nonresponse. The study used 43,150 weighted children. A multi-level logistic regression model was used, and *P* - values of ≤ 0.2 and < 0.05 were used to declare candidate variables in the binary, and multivariable to declare significant variables, respectively.

**Results:**

The prevalence of bottle feeding among children under-two-years-old in East Africa was 10.08% (95% CI 9.79, 10.36), ranging from 4.04% (95% CI 3.56, 4.53) in Tanzania to 33.40% (95% CI 32.72, 34.08) in Kenya. High antenatal care communities (AOR 1.22; 95% CI 1.11, 1.35), mothers aged 25–34 years (AOR 1.17; 95% CI 1.06, 1.28), high wealth index communities (AOR 1.12; 95% CI 1.02,1.25), women who had at least one types mass media exposure (AOR 1.64; 95% CI 1.53, 1.77), women from communities with high level mass media exposure (AOR 1.36; 95% CI 1.23, 1.52), given first birth after teenage years (AOR 1.17; 95% CI 1.09, 1.26), having more than one health visit in the year (AOR 1.37; 95% CI 1.27,1.47), multiple children (AOR 1.46; 95% CI 1.22, 1.75) were associated with higher rates of bottle feeding. Whereas a primary education (AOR 0.51; 95% CI 0.47, 0.54), having 3–5 living children (AOR 0.86; 95% CI 0.79, 0.95), a rural setting (AOR 0.53; 95% CI 0.49, 0.58), and a long distance from health facilities (AOR 0.84; 95% CI, 0.78, 0.91) were associated with lower rates of bottle feeding.

**Conclusions:**

The overall prevalence of bottle feeding was moderate in East African countries. Improving the availability and accessibility of health facilities to mothers, utilizing maternal healthcare, and media exposure will contribute to a significant decrease in the inappropriate bottle feeding of children in East Africa.

## Background

“Bottle feeding” is described by the World Health Organization (WHO) as feeding a baby or a young child aged 0–23 months any liquid (including breast milk) or semi-solid meal from a bottle with a nipple/teat [1]. Infant and young child feeding (IYCF) practices determine the nutritional condition of children under the age of two and have an impact on their survival, growth, and development [2, 3]. According to the WHO recommendations for age-appropriate breastfeeding for infants under the age of two, children aged zero–five months should be exclusively breastfed, while children aged six to twenty-three months should receive both breast milk and supplemental food [2, 4].

In Africa and other developing countries, the prevalence and duration of breastfeeding are decreasing, and bottle feeding has substantially replaced breastfeeding [5–7]. Furthermore, the 2022 Kenyan Demographic and Health Survey report shows that bottle feeding among children aged under two increased to 34% [8].This an indicator that bottle feeding has increased over time [9]. The two main factors that tend to shorten breastfeeding are urbanization and mothers’ higher educational level [7, 9].

Bottle feeding is one of the seven optional indicators used to assess IYCF practices [10]. It was used by 35.7% of children under the age of two in Namibia [11], and 12% in Ghana [12]. A greater incidence (38%) has been observed in Ethiopia’s Oromia region [13]. Because bottle feeding might increase the risk of excessive weight gain, malnutrition, iron deficiency, and decreased birth spacing, it is not recommended for children [14]. If fed by bottle, even expressed breast milk may increase newborn weight gain [14]. Avoiding the use of pacifiers or artificial teats is important for promoting universal breastfeeding [15]. Bottle feeding in newborns is closely linked to poor breastfeeding conditions [10, 15]. Studies have also shown that bottle feeding exposes children to childhood obesity and diabetes mellitus and increases their chance of developing gastrointestinal (GI) infections compared to exclusively breastfed children [16, 17].

The detrimental effects of bottle feeding are most severe in low-income settings and developing countries owing to a lack of access to clean water, sanitation, and hygiene, as well as a high percentage of illiteracy among mothers and guardians [18].

Although there were no specific explanations, the primary reasons for mothers to bottle feed their child were insufficient breast milk [19], ease of feeding the child [13, 20], stopping the child’s crying [19, 20], and promoting children’s growth [21]. Mothers’ and children’s ages [13, 20], as well as receiving counseling on the hazards of bottle feeding, had significant relationships with bottle feeding practice [13, 20]. To the best of our knowledge, no study has assessed determinants of bottle feeding and its prevalence in East Africa. The purpose of this study was to determine the prevalence of bottle feeding, and determinants among children aged 0–23 months in East Africa.

## Methods

### Study setting, period, and time frame

The data were obtained from the most recent standard DHS dataset of East African countries (2015/16–2022) (Table 1). A standardized dataset was used [22] to obtain all parameters and a large sample size that is representative of the population source. DHS collects data that is cross-nationally comparable. The surveys are population-based and nationally representative of each country, with large sample sizes [22]. Eastern Africa comprises 14 countries located in the Great Lakes region, the Horn of Africa, and the Indian Ocean Islands.

**Table 1:** Countries, sample size, and survey year of Demographic and Health Surveys included in the analysis for 10 East African countries

Country	Survey year	Weighted sample	Weighted frequency
Burundi	2016/17	5,341	12.38
Ethiopia	2016	4,203	9.74
Kenya	2022	3,397	7.87
Madagascar	2021	4,759	11.03
Malawi	2015/16	6,430	14.90
Rwanda	2019/20	3,221	7.46
Tanzania	2015/16	4,047	9.38
Uganda	2016	5,664	13.13
Zambia	2018	3,748	8.69
Zimbabwe	2015	2,339	5.42

### Data source and population

DHS databases for children’s records or child records were utilized. Before using the DHS dataset, weighted values were employed to restore the representativeness of the sample data. The source population included all children aged 0–23 months over the five years before the survey period in East Africa. Mothers who had more than one child within the previous two years were asked about the most recent or younger child. However, mothers who had twins in the previous birth were asked about both children [22]. This study covered all children aged 0–23 months in the five years preceding the survey in the selected enumeration areas (EAs) in each country. Children born recently and who died were excluded from the study. According to the DHS recode manual for the treatment of missing values, missing and “don’t know” replies on whether the child drank from a bottle with a nipple yesterday throughout the day or night were included in the study but were regarded as not using bottle feeding [22]. Finally, weighted 43,150 samples were analyzed.

### Sample size determination and sampling technique

Demographic and Health Survey (DHS) samples are frequently stratified by administrative geographic region and within each region by urban/rural areas. In the first round of sampling, the EAs were chosen with a probability proportional to their size within each stratum. The systematic sampling approach selected a predetermined number of households in the specified EAs in the second sampling step. Following the listing of the households, a fixed number of households were chosen in the designated cluster using equal-probability systematic sampling [22].

### Study variables

The bottle feeding practice of children aged 0–23 months was the outcome variable. Factors such as the mother’s age, work status, marital status, family size, maternal education, age at first birth, number of health facility visits, media exposure, household wealth status, child’s age, birth weight, breastfeeding status, sex, twins, place of delivery, pregnancy preference, birth order, preceding birth interval, distance to the health facility, Postnatal Care (PNC), and Antenatal Care (ANC), community-level factors, such as distance from health facilities, ANC, women’s education, mass media exposure, place of living, and community wealth level, were all assessed at the community level.

### Data processing and analysis

The DHS files for child record were downloaded in the STATA format. Following access to the data, they were cleaned, coded, and merged to provide suitable variables for analysis. The data were then weighted using sample weights for probability sampling and non-response to restore representativeness before statistical analysis. To define the variables in the study using statistical measurements, Microsoft Excel 2019 and STATA 17 software were used to provide both descriptive and analytic statistics.

### Model building for multi-level analysis

The usual logistic regression model assumptions may be violated due to the hierarchical nature of the DHS data. Consequently, a multilevel logistic regression with four models was fitted. The null model was used to evaluate variability in bottle feeding across clusters. The second model contained factors at the individual level, whereas the third model incorporated variables at the community level. In the final model (Model 4), both individual- and community-level variables were fitted simultaneously with the prevalence of bottle feeding. For model comparisons, the log-likelihood hood and deviation tests were utilized, and the model with the highest log-likelihood hood and lowest deviance value was chosen as the best-matched model. Variance inflation factor (VIF) was used to detect multicollinearity. All variables had VIF values of less than 10, with the final model’s mean VIF value being 1.46.

### Parameter estimation method

Furthermore, this model served as a litmus test to determine whether multilevel or conventional logistic regression should be used, justifying the employment of such a framework. It was assessed using the log-likelihood ratio test (LLR), median odds ratio (MOR), intraclass correlation coefficient (ICC), and proportional change of variance (PCV). Moreover, the model comparison was made using model deviance, with the model with the lowest deviance selected for reporting and interpreting results.

**Null model.** For individual *i* in community j, the model can be represented as [23, 24]:

$${Y_{ij}} + {\text{ }}\Upsilon 00 + {u_{0j}} + \varepsilon ij...........nullmodel $$

Where: Y_*ij*_ is the bottle feeding status of *i*^th^ child in the *j*^th^ cluster, µ_*00*_ = is the intercept; that is the probability of having bottle feeding in the absence of explanatory variables, µ_*0j*_ = community-level effect; ε_*ij*_ error at the individual level.

Mixed model: This model was derived by mixing both individual and community-level factors simultaneously [25].

$${Y_{ij}} + \Upsilon 00 + \Upsilon k0{X_{kij}} + {\text{ }}\Upsilon 0p{z_{pj}} + {\text{ }}{u_{0j}} + \varepsilon ij.{\text{ }}.{\text{ }}.{\text{ }}.{\text{ }}.{\text{ }}. $$

Where: The term γ_*k0*_ is the regression coefficient of the individual-level variable X_*k*_ and γ_*0p*_ is the regression coefficient of the community-level variable Z*p*. X_*k*_ and Z*p* were individual and community-level explanatory variables respectively. The subscripts *i* and *j* represent the individual level and cluster number respectively.

## Results

### Sociodemographic characteristics of the study participant

In this study, 43,150 weighted children aged 0–23 months were enrolled in East African countries. Regarding maternal characteristics, approximately half of the 19,098 (44.26%) study participants were between 25 and 34 years of age. Similarly, about 23,144 (53.44%) had average weight at birth (Table 2).

**Table 2:** Sociodemographic, maternal and child related characteristics on bottle feeding practice among 0–23 months old in East African countries recent DHS (weighted *n* = 43,150)

Multiple micro nutrient powder	Frequency(weighted)	Percentage
**Variables**		
**Maternal age**		
15–24	16,068	37.24
25–34	19,098	44.26
35–49	7,984	18.50
**Maternal education**		
Not educated	8,958	20.76
Primary	21,866	50.68
Secondary & higher	12,325	28.56
**Maternal employment**		
No	12,870	29.83
Yes	30,280	70.17
Wealth index		
Poorest	10,245	23.74
Poorer	9,226	21.38
Middle	8,394	19.45
Richer	8,071	18.70
Richest	7,214	16.72
**Mass media exposure**		
No	24,408	56.57
Yes	18,741	43.43
**Preceding birth interval in months**		
7–24	7,302	16.92
24–36	10,971	25.43
37–59	9,324	21.61
> 59	15,553	36.05
**Age at first birth**		
< 20	24,838	57.56
≥ 20	18,312	42.44
**Total children born**		
> 3	19,016	44.07
3–5	16,470	38.17
> 5	7,664	17.76
**Marital status**		
Never married	2,993	6.94
Married	29,322	67.95
Divorced/widowed	10,835	25.11
**Place of delivery**		
Home	10,092	23.39
Health facility	33,058	76.61
**At least one-time ANC follow-ups**		
No	2,543	5.89
Yes	40,607	94.11
**Number health visits**		
Once	33,093	76.69
More than one	10,057	23.31
**Distance to health facility**		
Not a big problem	25,374	58.80
A big problem	17,776	41.20
**Twin status**		
Single	41,993	97.32
Multiple	1,157	2.68
**Birth order**		
1st	10,458	24.24
2nd or 3rd	15,844	36.72
4th or above	16,847	39.04
**Child size**		
Small	7,734	17.92
Average	23,144	53.44
Large	12,272	28.24
**Child in months age**		
< 6	11,080	25.68
6–11	11,050	25.61
12–23	21,020	48.71
**Currently breastfeeding status**		
No	17,145	39.73
Yes	26,005	60.27
**Place of residence**		
Urban	8,643	20.03
Rural	34,507	79.97
**Community ANC coverage**		
Low	22,403	51.92
High	20,747	48.08
**Community education**		
Low	21,340	49.46
High	21,810	50.54
**Community mass media exposure**		
Low	22,020	51.03
High	21,130	48.97
**Community distance to health facility**		
Low	21,608	50.08
High	21,542	49.92
**Community wealth**		
Low	21,906	50.77
High	21,244	49.23

### Random effect model analysis

A significant variance in the chance of being exposed to bottle feeding was found in the null model (community-level variance = 0.40, *p* 0.001). Regional differences accounted for 10.84% of the variation in children’s use of bottle feeding, as shown by the ICC in the null model. Furthermore, the median OR was 1.83, which means that when children moved from a low to a high use of bottle feeding or intake prevalence area, the risk of being exposed to use of bottle feeding increased by 1.83 times. The PCV in this study was 47.5%, indicating that both community/country- and individual-level factors explained 47.5% of the national variation observed in an empty model.

### Fixed model analysis

Women aged 24–34 years had increased odds of bottle feeding compared to those aged 15–24 years (AOR 1.17; 95% CI 1.06, 1.28). Mothers from high community-level wealth were more likely to practice bottle feeding than those from poor communities (AOR 1.12; 95% CI 1.02, 1.25). However, mothers who completed primary education were less likely to engage in bottle feeding compared to women with secondary or higher-level education (AOR 0.51; 95% CI 0.47, 0.54). Higher ANC coverage in communities was associated with a 22% higher likelihood of bottle feeding (AOR 1.22; 95% CI 1.11, 1.35). Mothers with mass media exposure were more likely to practice bottle feeding (AOR 1.64; 95% CI 1.53, 1.77), as were those living in communities with high mass media exposure (AOR 1.36; 95% CI 1.23, 1.52). Mothers with 3–5 living children had lower odds of bottle feeding compared to those with fewer than three children (AOR 0.86; 95% CI 0.79, 0.95). Giving birth to the first child after the teenage years increased the odds of bottle feeding (AOR 1.17; 95% CI 1.09, 1.26). More health facility visits were associated with higher odds of bottle feeding (AOR 1.37; 95% CI 1.27, 1.47). Multiple children were more likely to receive bottle feeding compared to single children (AOR 1.46; 95% CI 1.22, 1.75). Children aged 6–11 months and 12–23 months had higher odds of bottle feeding compared to children less than six months old (AOR 2.67; 95% CI 2.42, 2.94 and AOR 1.85; 95% CI 1.69, 2.02, respectively). Mothers from rural areas had lower odds of bottle feeding (AOR 0.53; 95% CI 0.49, 0.58), as did those reporting distance to a health facility as a major problem (AOR 0.84; 95% CI 0.78, 0.91) (Table 3).

**Table 3:** Individual and community-level factors independently associated with bottle feeding among 0–23 months old children in east Africa (weighted *n* = 43,150)

Bottle feeding			Null model	Model I	Model II	Model III
Dependent Variables	No, n (%)	Yes, n (%)	AOR (95%CI)	AOR (95% CI)	AOR (95% CI)	AOR (95% CI)
**Maternal age**						
15–24	14,595(90.83)	1,473(9.17)		1		1
25–34	16,969(88.85)	2,129(11.15)		1.25(1.14,1.37)		1.17(1.06,1.28) *
35–49	7,239(90.67)	745(9.33)		1.15(1.01,1.32)		1.06(0.92,1.21)
**Maternal education**						
No education	8,334(93.33)	625(6.97)		0.77(0.70,0.86)		0.91(0.81,1.01)
Primary	20,417(93.37)	1,449(6.63)		0.45(0.41,0.48)		0.51(0.47,0.54) *
Secondary/higher	10,052(81.56)	2,273(18.41)		1		1
**Mass media exposure**						
No	22,870(93.70)	1,538(6.30)		1		1
Yes	15,932(85.01)	2,809(14.99)		1.84(1.71,2.98)		1.64(1.53,1.77) *
**Total children born**						
> 3	16,765(88.17)	2,250(11.83)		1		1
3–5	14,928(90.64)	1,542(9.36)		0.83(0.76,0.91)		0.86(0.79,0.95) *
≥ 6	7,110(92.76)	555(7.24)		0.82(0.71,0.94)		0.89(0.78,1.03)
**Marital status**						
Never married	2,621(87.57)	372(12.73)		1		1
Married	26,320(89.76)	3,002(10.24)		1.06(0.89,1.21)		1.06(0.93,1.21)
Divorced/widowed	9,862(91.02)	973(8.98)		0.87(0.76,1.00)		0.88(0.77,1.02)
**Place of delivery**						
Home	9,436(93.50)	656(6.50)		1		1
Health facility	29,367(88.84)	3,691(11.16)		1.14(1.04,1.24)		1.05(0.96,1.15)
**Age at first birth**						
< 20	22,801(91.80)	2,037(8.20)		1		1
≥ 20	16,002(87.38)	2,310(12.62)		1.16(1.08,1.25)		1.17(1.09,1.26) *
**Number of health visits**						
Once	29,946(90.49)	3,148(9.51)		1		1
More than one	8,857(88.08)	1,199(11.92)		1.39(1.29,1.49)		1.37(1.27,1.47) *
**Twin status**						
Single	37,837(90.10)	4,156(9.90)		1		1
Multiple	966(83.50)	191(16.50)		1.48(1.24,1.77)		1.46(1.22,1.75) *
**Child size**						
Average	20,728(89.56)	2,416(10.44)		1		1
Large	11,123(90.64)	1,149(9.36)		0.91(0.85,0.99)		0.92(0.85,0.98) *
Small	6,952(89.89)	782(10.11)		1.07(0.98,1.17)		1.09(0.99,1.20)
**Child in months age**						
> 6	10,353(93.44)	727(6.56)		1		1
6–11	9,538(56.32)	1,512(13.68)		2.64(2.40,2.91)		2.67(2.42,2.94) *
12–23	18,911(89.97)	2,109(10.03)		1.83(1.67,2.01)		1.85(1.69,2.02) *
**Child sex**						
Male	19,470(90.01)	2,160(9.99)		1		1
Female	19,332(89.84)	2,187(10.16)		0.96(0.90,1.02)		0.96(0.90,1.02)
**Distance to health facility**						
Not big problem	22,317(87.95)	3,056(12.05)		1		1
Big problem	16,486(92.74)	1,291(7.26)		0.75(0.70,0.81)		0.84(0.78,0.91) *
**Residence**						
Urban	6,941(80.30)	1,702(19.70)			1	1
Rural	31,862(92.34)	2,645(7.66)			0.36(0.34,0.39)	0.53(0.49,0.58) *
**Community level Wealth**						
Low	19,279(88.01)	2,627(11.99)			1	1
High	19,524(91.90)	1,720(8.10)			1.17(1.04,1.31)	1.12(1.02,1.25) *
**Community level distance to health facility**						
Low	19,123(88.50)	2,484(11.50)			1	1
High	19,680(91.35)	1,863(8.65)			0.85(0.77,0.95)	0.91(0.83,1.01)
**Community ANC coverage**						
Low	20,390(91.02)	2,012(8.98)			1	1
High	18,413(88.75)	2,335(11.25)			1.31(1.18,1.46)	1.22(1.11,1.35) *
**Community media exposure**						
Low	20,297(92.18)	1,722(7.82)			1	1
High	18,505(87.58)	2,625(12.42)			1.60(1.42,1.78)	1.36(1.23,1.52) *
**Random parameters and model comparison**						
Community level variance			0.40	0.22	0.33	0.21
ICC (%)			10.84	6.40	8.99	5.81
Median OR (%)			1.83	1.56	1.73	1.55
PCV (%)			Reference	45	17.5	47.5
LLR			-14622.76	-13625.46	-14127.42	-13464.05
DIC			29,245.52	27,250.92	28,254.84	26,928.1
AIC			29249.53	27290.92	28268.83	27194.94

Note: * significant at *p*-value < 0.05, ICC = Intra cluster correlation, MOR = Median Odds Ratio, DIC = Deviance information criterion, LLR = Log Likelihood Ratio, AIC = Akaike information criterion

### Prevalence of bottle feeding among children in East Africa

**Fig. 1:**
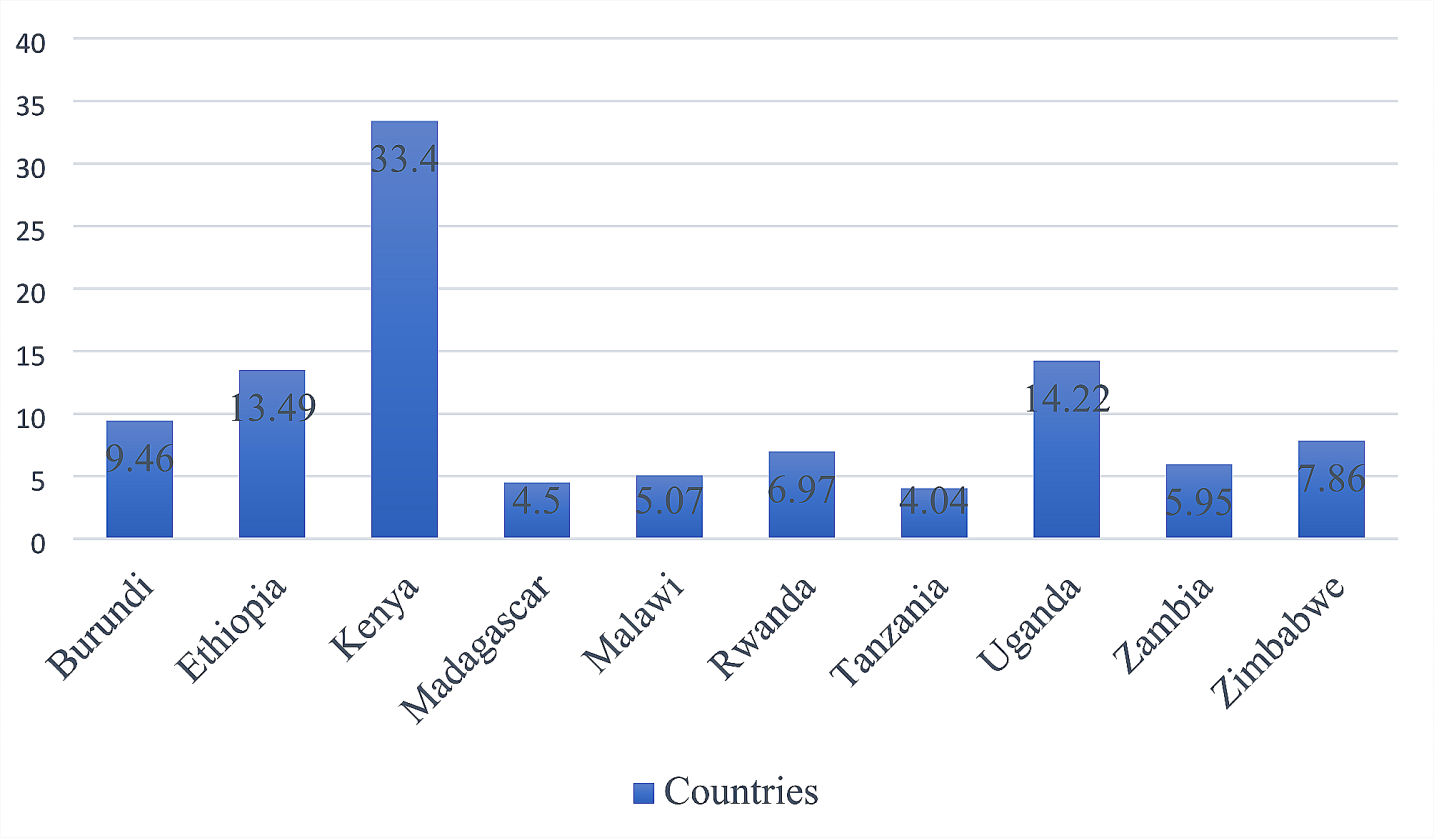
Percentage of children 0–23 months of age who are fed with a bottle*. *Defined as “(children 0–23 months of age who were fed with a bottle during the previous day/children 0–23 months of age) x 100”

The overall prevalence of bottle feeding among 0-23-month-old children in East Africa was found to be 10.08% (95% CI 9.79, 10.36). The highest prevalence of bottle feeding was in Kenya with 33.4%, and the lowest was in Tanzania with 4.04%. Uganda and Ethiopia scored more than 10%; however, Madagascar scored less than 5% (Fig. 1).

## Discussion

In this study, the prevalence of bottle feeding practice among children under the age of two years was 10.08%. This figure is lower than rates found in studies undertaken in Indonesia [26], Ethiopia [20], and Eastern Sudan [27]. This disparity could be attributed to differences in the sociocultural features of study participants, such as different cultural child feeding practices, time frame differences, analysis approach, and settings. Mothers older were more likely to use bottle feeding than young mothers. According to the search results, the practice of bottle feeding grows as mothers’ ages rise owing to a variety of factors such as perceived insufficient breast milk production, being overworked, sluggish infant growth, and a lack of understanding about the benefits of nursing [28]. Various studies demonstrate that when the mother’s age increases, there is for high tendency of high prevalence of intention to bottle feeding in the region, which could be linked to a reduced degree of understanding about bottle feeding [29]. This same cause was stated in other investigation [29]. These mothers should be aware that breastfeeding cessation, particularly within the first half-year of life or shifting to bottle feeding, is a major risk factor for infant and childhood illness and mortality. Similarly, regarding the number of living children, the search results offer data on the prevalence of and factors associated with bottle feeding practices with infants under the age of two. It was discovered that having 2–5 children was substantially connected with bottle feeding practice [30]. This may be related to older mothers and the resource shortage mentioned in the previous sections.

The findings of this study show that as women’s education levels rise, so does their child’s use of bottle feeding. This is consistent with research from Indonesia [26], Ethiopia [20], and Namibia [11]. Educated mothers may have a busier work schedule than housewives (no paid work), and they may not have the time to breastfeed [26]. The study’s findings regarding mothers’ educational status and bottle feeding demonstrate that a higher mother’s educational status does not necessarily imply improved awareness and understanding of the benefits of nursing [11]. Children from wealthy families are more likely to be bottle fed than children from low-income families. Research conducted in Namibia [11], Ethiopia [20], and Indonesia [26], supports this. This could be because wealthy families have access to other feeding options such as nipple or bottle feeding [26]. This conclusion could be explained by the fact that mothers in the higher wealth quintile may have easy access to more expensive feeding options, which could affect their decision to bottle feed.

The odds of bottle feeding increases with the child’s age. A study conducted in Ethiopia [20], Namibia [11], and Indonesia [26] found that older children were more likely to use bottle feeding than youngsters. This is due to the fact that as children grow older, they may have more feeding options, such as drinking water, tea, and processed milk, which may result in a higher rate of bottle feeding [26]. Mothers with such prior experience may be less likely to start bottle feeding their child at a young age [30]. Women who are urban, educated, or empowered are more likely to attend health facilities while pregnant [31]. Compared with a child whose birthweight was normal, a child who was heavier demonstrated a lower likelihood of bottle feeding. It is unlikely that mothers themselves will be knowledgeable about high weight gain during infancy being connected to obesity in later life. It is more likely that mothers supplement a low-weight baby because they perceive that the baby is hungry [32, 33]. This connection may be explained by the fact that formula-fed newborns are always overfed.

In this study, exposure to mass media was found to have a beneficial association with bottle feeding. Media exposure can have an impact on bottle feeding practices. According to studies, media coverage frequently portrays bottle feeding as easier and more common than nursing, which can have a negative impact on the prevalence of breastfeeding [34]. Rural women were less likely to bottle feed than their counterparts. This finding is consistent with prior research [11]. A plausible explanation for this result could be that most urban mothers came from families with higher socioeconomic status than their rural counterparts, which may have facilitated their access to breast milk substitutes and information on breast milk substitutes. Additionally, most urban mothers are likely to have paid employment, and the pressure to return to work after maternity leave may result in bottle usage [20].

The use of nationally representative data with a large sample, which makes it representative at the country level, was the study’s key strength to generalize the estimates. As the data were gathered cross-sectionally through self-reported interviews, they were subject to recall and social desirability bias. Furthermore, although, the time interval was short and may have a minimal effect on the study outcome, time was not considered as an independent variable.

## Conclusions

The overall bottle feeding practice as compared to past years though it may well seem moderate compared to rates of bottle feeding in Europe and North America. The findings indicate that each country’s ministry of health, policymakers, implementers, and other stakeholders should prioritize avoidable variables such as educating women in optimal breastfeeding practices, and reducing bottle feeding practices in the region.

## Data Availability

Not applicable.
